# Trueness of five different 3D printing systems including budget- and professional-grade printers: An In vitro study^☆^

**DOI:** 10.1016/j.heliyon.2024.e26874

**Published:** 2024-02-23

**Authors:** Dénes Palaszkó, Anna Németh, Gréta Török, Bálint Vecsei, Boldizsár Vánkos, Elek Dinya, Judit Borbély, Gyula Marada, Péter Hermann, Barbara Kispélyi

**Affiliations:** a Department of Prosthodontics, Faculty of Dentistry, Semmelweis University, Budapest, Hungary; b Institute of Digital Health Sciences, Semmelweis University, Budapest, Hungary; c University of Pécs, Hungary

**Keywords:** CAD-CAM, 3-D Printing, Model, Rapid prototyping, Digital dentistry

## Abstract

**Problem:**

Several types of 3D printers with different techniques and prices are available on the market. However, results in the literature are inconsistent, and there is no comprehensive agreement on the accuracy of 3D printers of different price categories for dental applications.

**Aim:**

This study aimed to investigate the accuracy of five different 3D printing systems, including a comparison of budget- and higher-end 3D printing systems, according to a standardized production and evaluation protocol.

**Material and methods:**

A maxillary reference model with prepared teeth was created using 16 half-ball markers with a diameter of 1 mm to facilitate measurements. A reference file was fabricated using five different 3D printers. The printed models were scanned and superimposed onto the original standard tesselation language (.stl) file, and digital measurements were performed to assess the 3-dimensional and linear deviations between the reference and test models.

**Results:**

After examining the entire surface of the models, we found that 3D printers using Fused filament fabrication (FFF) technology −120.2 (20.3) μm create models with high trueness but high distortion. Distortions along the z-axis were found to be the highest with the stereolithography (SLA)-type 3D printer at −153.7 (38.7) μm. For the 4-unit FPD, we found 201.9 (41.8) μm deviation with the digital light processing (DLP) printer. The largest deviation (−265.1 (55.4) μm) between the second molars was observed for the DLP printer. Between the incisor and the second molar, the best results were produced by the FFF printer with −30.5 (76.7) μm.

**Conclusion:**

Budget-friendly 3D printers are comparable to professional-grade printers in terms of precision. In general, the cost of a printing system is not a reliable indicator of its level of accuracy.

## 1 Introduction

Computer-aided design/computer-aided manufacturing (CAD-CAM) systems have evolved enormously in recent years, particularly in the field of dentistry, enabling accurate and time-saving manufacturing. CAM technologies use a manufacturing machine connected to a computer to produce virtually designed object [1]. CAM can be divided into subtractive and additive manufacturing processes. Subtractive methods use cutting devices in which a workpiece is subtracted from a prefabricated block to produce the desired plan [2]. One disadvantage is that the process is wasteful because the remaining material cannot be reused [3].

By contrast, additive manufacturing (AM), also known as rapid prototyping or 3D printing, wastes significantly less material [57]. The American Society for Testing and Materials has defined AM as “a process of joining materials to make objects from 3D model data, usually layer upon layer, as opposed to subtractive manufacturing methodologies” [4]. Additive manufacturing also facilitates the creation of complex shapes, hollow geometries, and undercut areas to easily be created. This contrasts with milling, which imposes limitations on the cutting device and cutting angle [5].

Because additive production is time- and material-efficient, and manufacturing is more sustainable, 3D printing is becoming more appealing for dental applications [6,7]. Dentists and technicians can print different dental devices for orthodontic applications such as clear aligners from biocompatible and dimensionally stable clear resins [8]. For surgical purposes, the most commonly used devices are surgical guides, which promise improved implant placement accuracy and precision compared with conventional non-guided implant surgery [9,10]. 3D printing is most commonly used in the field of prosthodontics because several tools and prostheses can be produced with this technology, e.g. custom trays, temporary and permanent fixed restorations, and complete dentures [11,12,58].

Dental models are used in several fields of dentistry. They can be used for preoperative planning and training before oral or maxillofacial surgery [13], whereas physical models are required to produce orthodontic appliances such as trays for indirect bracket bonding or clear aligners [14]. From a prosthodontic perspective, one of the biggest advantages of CAD-CAM technology and digital workflow is that a physical model is not required to produce a fixed restoration because it can be designed digitally, onscreen, with dental software applications, and fabricated with computer-aided production such as milling or 3D printing [15]. Despite the virtual workflow, there are digitally designed and implemented cases in which a physical model is necessary to adjust the restoration, for example, for veneering or contact point correction [16]. Both dental-supported and implant-supported restoration fabrication require definitive casts that can be mounted on articulators to evaluate occlusion and articulation and adjust approximal contacts [17]. In these cases, printing accuracy is crucial because if the preparation line or the implant location is inaccurate, the prosthesis made from it will not fit precisely and will be unusable and unwearable [17].

Owing to increasing market development, several types of 3D printers are now available, employing various manufacturing technologies and materials, and at various price points. Hence, reliable information regarding the differences between budget and higher-end printing systems, particularly in terms of accuracy, is required.

In dental model fabrication, the most used technologies are the vat-polymerization techniques stereolithography and digital light processing using various resins [18]. The main settings and steps of the printing workflow are printing parameters (e.g., layer thickness), support parameters, slicing methods, and post-processing procedures, which significantly affect the characteristics of the final product [20]. During SLA, which was the earliest 3D printing technology, the building platform is immersed in photosensitive liquid resin and then cured using a UV laser. The build platform is then elevated to an amount equivalent to the layer thickness, and the subsequent layer is polymerized. This is repeated until the entire object is built.^19^ During DLP, the printing process is similar; however, the light source is a projector based on a microelectromechanical system that utilizes a digital mirror device [20]. Liquid crystal display (LCD) printers also belong to the vat-polymerization category, but they use an LCD as an imaging system to generate a mask and block light from the light-emitting diode (LED) back panel [21]. Fused filament fabrication involves the extrusion of thermoplastic materials (filaments of plastic or metal wire) through a heated nozzle that can be adjusted horizontally and vertically [22].

Despite the variety of techniques and application opportunities, there is an unmet need to evaluate the accuracy of this manufacturing method because the results in the literature are not consistent on this topic. Kim et al. compared the accuracy of models printed using four techniques and found that DLP techniques were more precise than SLA and FFF [23]. In another study, conventional, milled, and two types of printed dental models were evaluated, and the SLA models showed better trueness than the DLP models for all prepared teeth, except for the inlay preparation [24]. Maura et al. [25] conducted a cross-sectional study to analyze the trueness and precision of dental models produced using SLA and DLP printers. The results showed that the SLA printer demonstrated significantly higher precision and trueness than the DLP printer.

The differences in results between studies can be caused by several factors, such as the material used [26], layer thickness [27], support structures [28], building angle [29,30], model design [31], post-processing procedures [30], slicer software [32], storage conditions [32], and other printing parameters, such as lifting height and lifting speed [33,34]. This explains the difficulty in determining the accuracy of the printing process. For example, when objects are printed with different layer thicknesses using different types of materials and stored under different conditions, a comparison is not feasible, as it cannot be determined which factors cause the observed differences in accuracy. Common evaluation criteria and a defined printing protocol are required to accurately compare various printers and printed models. The parameters that can be controlled during production (e.g., layer thickness, model design, building angle) must be specified for a standardized comparison.

The aim of the present study was to investigate the accuracy of five different 3D printing systems, including a comparison of budget- and higher-end 3D printing systems, according to a standardized production and evaluation protocol. The null hypothesis was that there would be no significant difference in the accuracy between the models produced by the examined printers.

## 2 Material and methods

### 2.1 Generation of the digital master model

**Fig. 1:**
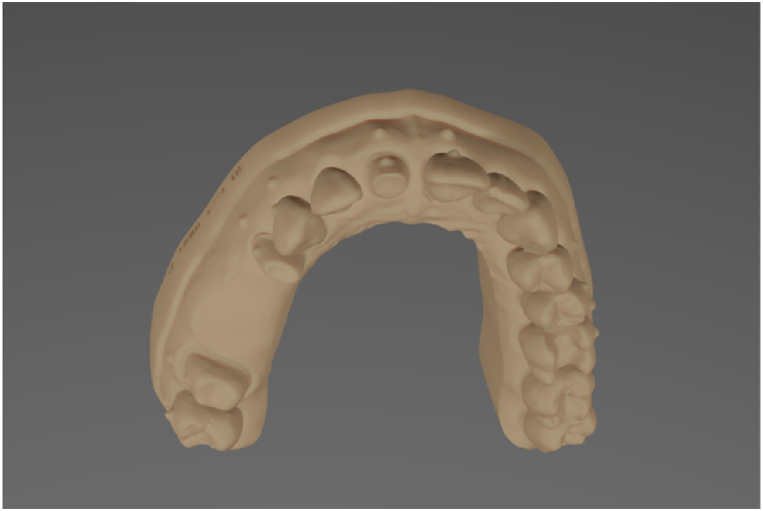
The digital image of the master model.

For this experiment, a maxillary model of a patient with partial edentulism was chosen. Four teeth were prepared in vitro (maxillary right second molar and maxillary right first premolar for a four-unit fixed partial denture [FPD]), maxillary left central incisor for a single crown, and maxillary left first molar for an inlay) and a master stereolithographic format (standard tesselation language, .stl) file was generated as a reference model for this study. To create reference point landmarks, thereby making point-based measurements more accurate and clear, sixteen 1-mm diameter half-ball markers were virtually generated and placed on the model. Eight markers were placed on the vestibular surface of the maxillary right third molar, maxillary right second molar, maxillary right first premolar, maxillary right central incisor, maxillary left central incisor, maxillary left second premolar, and maxillary left first molar, and eight markers were placed on the gingival part of the model in accordance with the abovementioned markers. The models were made solid with a flat horseshoe-shaped base without additional supporting structures, and 10 numbered models (from 1 to 10) were exported as.stl files. For the model building and modification, Model Maker (3Shape; Copenhagen, Denmark) software (version 2022.1) was used (Fig. 1).

### 2.2 Production of test models

Each prepared.stl file was printed using five different 3D printing units, yielding a total of fifty models. The 3D printing units used in this study were Pro4K80 (Asiga; Alexandria, Australia), Flow IDEX (CraftBot; Budapest, Hungary), Form 3B (Formlabs; Massachusetts, USA), Sonic 4K (Phrozen; Hsinchu, Taiwan), and D20+ (Rapidshape; Heimsheim, Germany). The Pro4K80 and D20+ printers utilize DLP technology, Flow IDEX utilizes fused deposition modeling (FDM) technology, Form 3B utilizes SLA technology, and Sonic 4K utilizes LCD technology. The basic characteristics and abbreviations of the printing units are listed in Table 1.

**Table 1:** Group characteristics.

Group	As	Cb	Fl	Ph	Rs
Printer	Pro 4K80	Flow Idex	Form 3B	Sonic 4K	D20+
Recommended Retail Price (USD)	24.999.-	3.399.-	7.569.-	1.699.-	12.305.-
Technology	DLP	FDM	SLA	LCD	DLP
Material	Pro3Dure Gr13	Polylite PETG	Model v3	Harz Labs Model Grey	Dreve Fotodent Model 2
Ambient temperature at printing site	19 °C	20 °C	22 °C	21 °C	20 °C
Humidity at printing site	29%	24%	23%	29%	23%
Slicing software	Composer	IdeaMaker	PreForm	Chitubox	Netfabb

**Fig. 2:**
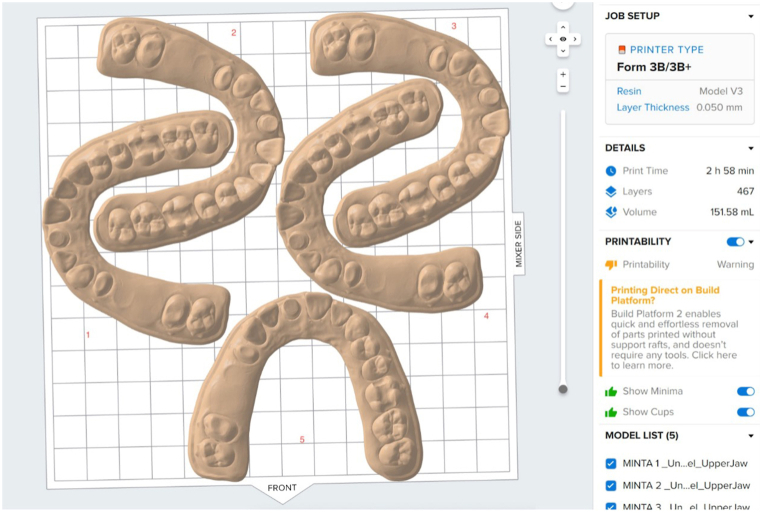
Positioning of the models in PreForm.

The models were oriented with the base parallel to the platform and positioned directly on the platform without any supporting structures (Fig. 2).

The slicing software recommended by the manufacturer was used for each printer. The names of the slicing software used are listed in Table 1.

The models were printed using five different photosensitive resins (one selected for each printer, as recommended by the manufacturer) marketed as dental model materials. The materials used for each printer are listed in Table 1.

Each printer was calibrated according to the manufacturer's instructions (except for Form 3B, for which calibration was unnecessary), and the printing parameters (layer height, printing speed, separation pressure limit, material viscosity, material hardness, resolution, and slice thickness) were set according to the values coded in the printing profiles of the materials.

The materials were mixed using a mixing machine in Group AS, hand-mixed in groups PH and RS, and no mixing was required in Group FL because the Form 3B printer has a built-in material mixer. At each printing site, the materials were inspected after mixing, and the presence or absence of air bubbles was recorded. The ambient temperature at the printing sites was between 19 °C and 22 °C, and the humidity was between 23% and 29%. The exact values can be found in Table 1.

### 2.3 Post-processing of the models

After printing, post-processing procedures were performed for the four groups according to the manufacturers’ instructions. Group CB (Flow Idex) did not require post-processing because Flow Idex does not utilize post-processing.

In Group AS (Pro4K80), the models were washed in an ultrasonic isopropyl immersion cleaning unit (Ultrasonic Cleaner, Soundlink; Shuzhou, China) for 6 min, followed by a water immersion bath (CLD1, Pro3Dure Medical; Iserlohn, Germany) for 7 min. After the washing procedure, the models were air-dried at room temperature with a compressed air duster and polymerized for 4 min at 22 °C using CLD2 (Pro3Dure Medical; Iserlohn, Germany). The first 40 s of post-curing involved nitrogen insufflation.

In Group FL (Form 3B), the models were washed in an isopropyl immersion bath using a Form Wash device (Formlabs; Massachusetts, USA) for 10 min. After cleaning, the models were air-dried at room temperature using a compressed air duster. The drying was followed by a 10-min polymerization at 60 °C using the Form Cure (Formlabs; Massachusetts, USA) curing machine.

**Fig. 3:**
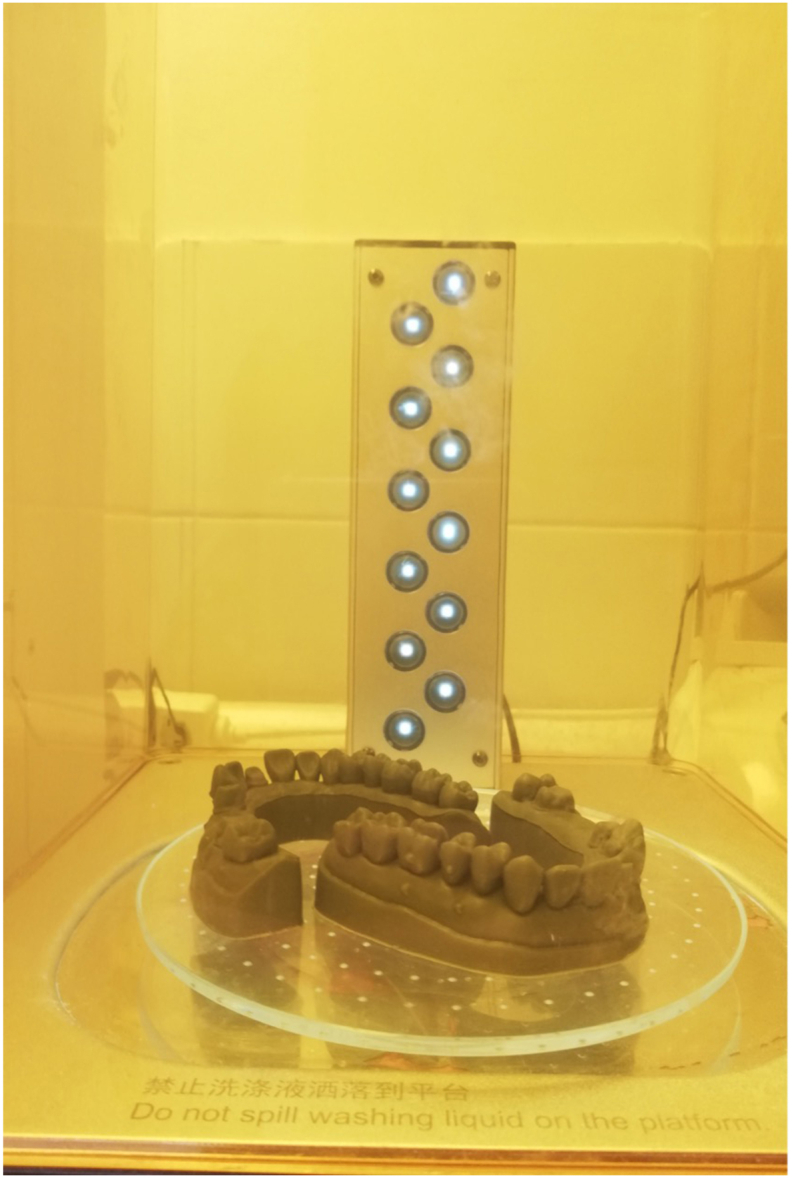
Post-processing using the Anycubic Wash And Cure 2.0.

In Group PH (Sonic 4K), the models were washed in isopropyl alcohol for 10 min and polymerized for 8 min at 60 °C utilizing a combined washing and curing device (WASH AND CURE 2.0, Anycubic; Hong Kong, China) (Fig. 3).

In Group RS (D20 +), the models were washed in an ultrasonic immersion bath (Ultrasonic Cleaner, ASonic; Nagytarcsa, Hungary) for 2 min followed by a water rinse. After air-drying with a compressed air duster at room temperature, the models were polymerized in a PCU LED (Dreve ProDiMed; Unna, Germany) post-curing device for 8 min at room temperature.

### 2.4 Transportation, handling, and storage of the finished printed models

The 3D-printed models were transported to the laboratory and stored by our workgroup in a hard-shell padded suitcase to ensure protection from physical impact and light.

Each group was assigned a dedicated storage drawer in which the models were stored widely spaced and flat on their bases. The ambient temperature of the storage room was between 20 °C and 23 °C and the humidity was between 52% and 60%.

### 2.5 Digitizing the finished printed models

Every model was digitized within one week using a Vinyl Open Air desktop scanner (Smart Optics; Bochum, Germany). The reference scanning procedure was performed by a trained technician (A. B.) at John von Neumann University at Kecskemét. After digitization of the models, the.stl files were imported into Geomagic Control X software (3D Systems, California, USA) to perform linear measurements between the markers and surface accuracy measurements.

### 2.6 Dimensional measurements

Accuracy can be described in terms of two factors: trueness and precision. Trueness concerns the ability to reproduce a virtual design as close to its real or true form as possible without distortions, and precision concerns the consistent reproduction of objects manufactured under the same conditions; that is, how identical the objects are to each other [35,36]. In this study, trueness of the measurements was analyzed using the root mean square (RMS) method.

To assess the overall accuracy of the printed surfaces, the models from each printer were superimposed onto the reference.stl file using the best-fit alignment method. Furthermore, a combination of the distances between the aforementioned reference points was used to assess the accuracy of the models in clinically important areas.

The entire deviation was calculated for surface reliability by selecting the teeth and gingival parts of the dental arch above the base of the model. The RMS values were exported for statistical analysis.

The horizontal and vertical linear dimensions were measured between the reference marker points in a given plane to evaluate the reliability of the printed objects.

**Fig. 4:**
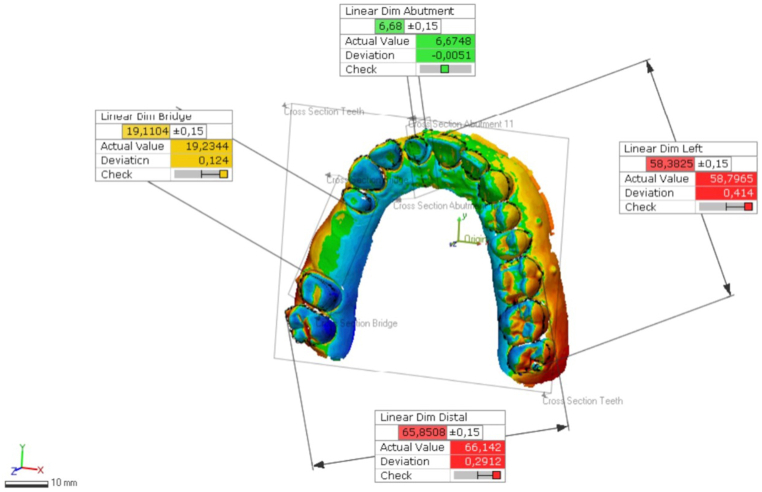
The following distances were measured digitally on the cast.

**Fig. 5:**
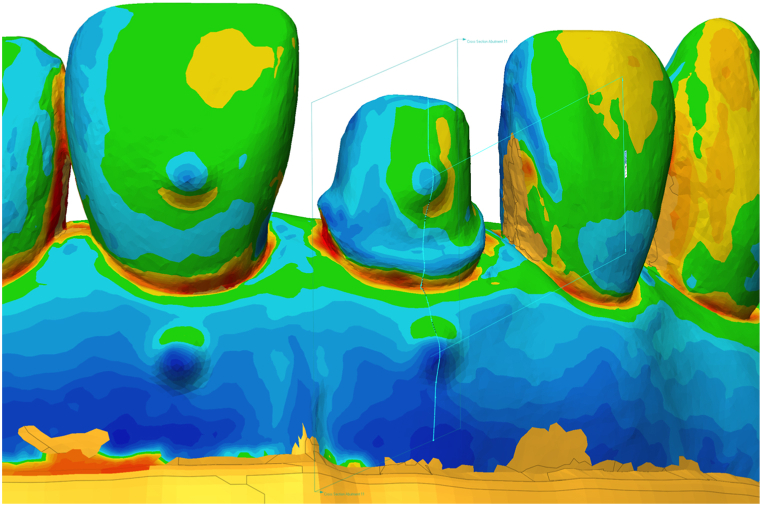
The vertical “Z” axis deviation measured between marks on right central incisor die and gingival surface.

The following dimensions were calculated (Fig. 4, Fig. 5): (1) the height of the prepared die - the distance between the reference marker points on the buccal surface of the upper right central incisor and a reference marker point on the gingival area of the central incisor; (2) the distance between the reference point on the buccal surface of the upper left central incisor and a reference point on the buccal surface of the upper left third molar; (3) the distance between the reference point on the buccal surface of the upper right first premolar and a reference point on the buccal surface of the upper right second molar (distance between abutments of a four-unit FPD); and (4) the distance between the reference point on the buccal surface of the upper right third molar and a reference point on the buccal surface of the upper left first molar.

### 2.7 Statistical analysis

For continuous data, results were characterized using descriptive statistics. Due to the small sample size, we also used the bootstrap procedure [55]. This process allowed us to calculate the standard errors and construct confidence intervals. The normality of the examined variables was checked using the Shapiro-Wilk test. Levene's test was used to check the homogeneity of the variances between groups. Continuous variables between printer subgroups were compared using Kruskal-Wallis analysis of variance with Bonferroni correction to determine the significance between pairs of groups.

The analysis was two-sided with a significance level of α = 0.05. Statistical analyses were performed using IBM SPSS (version 28.0; IBM Corporation, Armonk, NY, USA).

## 3 Results

Data from the virtual measurements were analyzed using descriptive statistics (mean and standard deviation [SD]) for each printer.

**Fig. 6:**
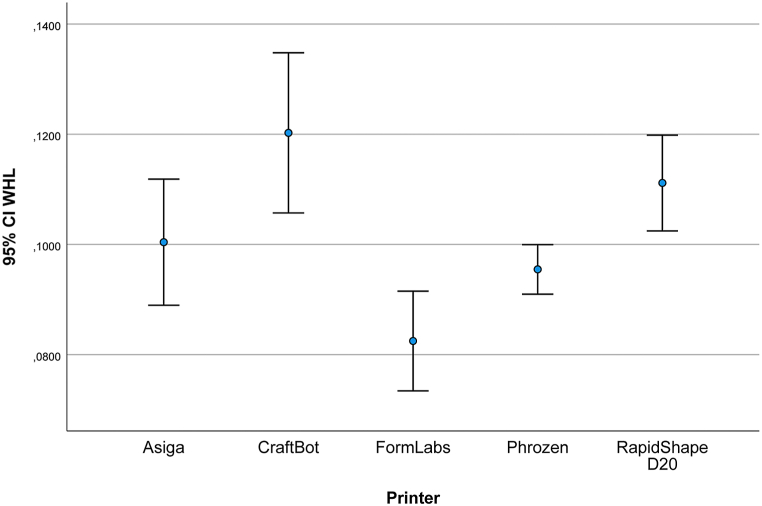
Whole deviation (WHL, μm).

Fig. 6 and Table 2 show the overall deviations of the models compared to the reference model. The mean (SD) was 100.39 (15.9) μm for the AS models, 120.2 (20.3) μm for the CB models, 82.4 (12.6) μm for the FL models, 95.4 (6.2) μm for the PH models and 111.1 (12.1) μm for the RS models. Table 2 shows the mean, SD, and lower and upper bounds of measured data in micrometers for each group compared to the reference data.

**Table 2:** Whole deviation (WHL, μm).

	Mean	Std. Dev.	95% Confidence Interval	
WHL			Lower	Upper
AS	100,390	15,9768	90,714	110,378
CB	120,240	20,3298	108,121	133,066
FL	82,470	12,6563	75,514	90,519
PH	95,460	6,2649	91,822	99,239
RS	111,130	12,1489	104,085	118,466

**Fig. 7:**
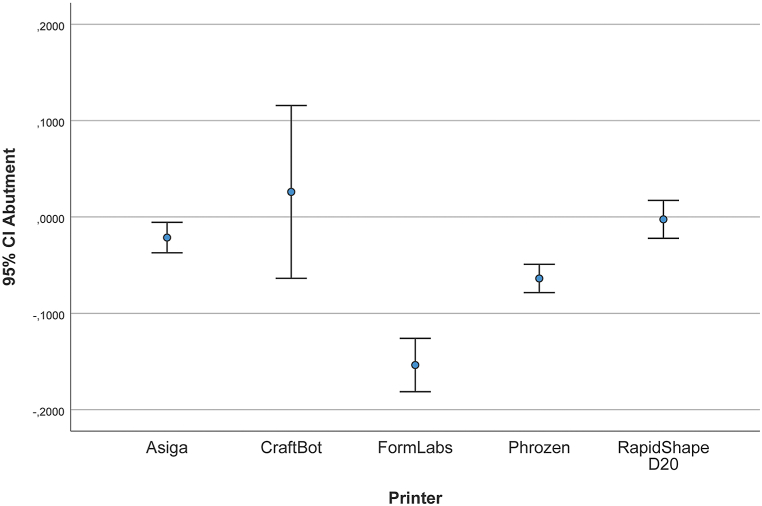
Deviation in height of the prepared right central incisor (μm).

Table 3 and Fig. 7 illustrate the measurement of the height of the prepared die, that is, the deviations that can be measured on the z-axis of the 3D models. Deviations between the test groups and the reference model were measured. The mean (SD) was −21.4 (22.1) μm for the AS models, 25.9 μm (125.5) for the CB models, −153.7 (38.7) μm for the FL models, 63.9 μm (20.5) for the PH models, and 2.4 μm (27.5) for the RS models.

**Table 3:** Linear deviation of different measurements (μm).

a) Deviation in distance between height of the prepared die				
	Mean	Std. Dev.	95% Confidence Interval	
			Lower	Upper
AS	−21,430	22,1063	−34,376	−8434
CB	25,940	125,3801	−44,357	105,137
FL	−153,730	38,7657	−177,690	−131,971
PH	−63,910	20,5899	−76,434	−51,962
RS	−2480	27,5444	−18,214	13,659
*b) Deviation in distance between 4-unit bridge abutments*				
AS	165,610	21,3623	152,985	178,562
CB	111,710	82,7347	67,319	169,754
FL	171,750	33,6906	150,114	190,782
PH	−28,280	23,1833	−41,494	−14,053
RS	201,970	41,8103	176,133	225,409
*c) Deviation in distance between right central incisor and left third molar*				
AS	431,410	68,2806	383,301	467,369
CB	−30,560	76,7037	−78,964	12,409
FL	189,370	55,5029	156,349	221,413
PH	−105,600	42,9296	−132,862	−80,914
RS	37,550	58,9854	2643	70,368
*d) Deviation in distance between upper right third molar and upper left first molar*				
AS	231,090	88,1928	174,925	283,548
CB	93,690	135,2049	6524	170,323
FL	51,030	70,9084	11,665	94,933
PH	−265,170	55,4311	−299,330	−234,473
RS	66,540	50,1374	39,009	97,299

Table 3 presents the results of the linear measurement points at different distances.

**Fig. 8:**
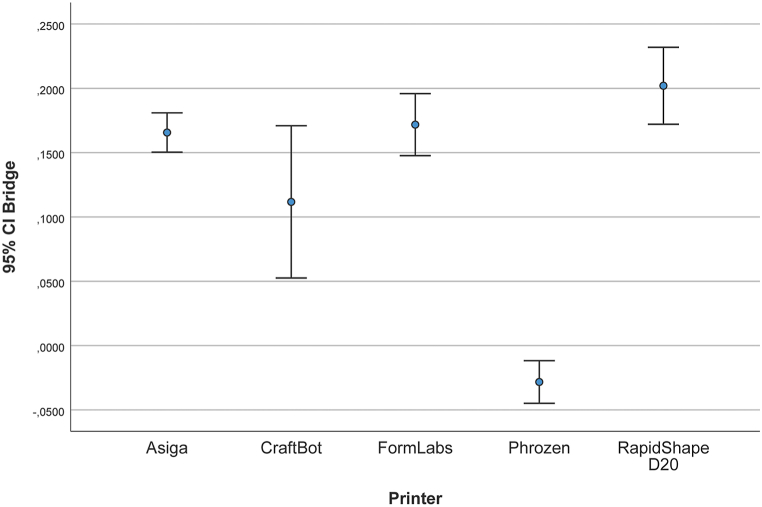
Deviation in distance between 4-unit FPD abutments (μm).

Table 3 and Fig. 8 show the mean, SD, and lower/upper bound of the measured data in micrometers. Measurements were performed between the upper right first premolar and the second molar. The following values show linear deviations compared with the reference measurements. The mean (SD) was 165.6 μm (21.3) for the AS models, 111.7 μm (82.7) for the CB models, 171.7 μm (33.6) for the FL models, −28.2 μm (23.1) for the PH models, and 201.9 μm (41.8) for the RS models.

**Fig. 9:**
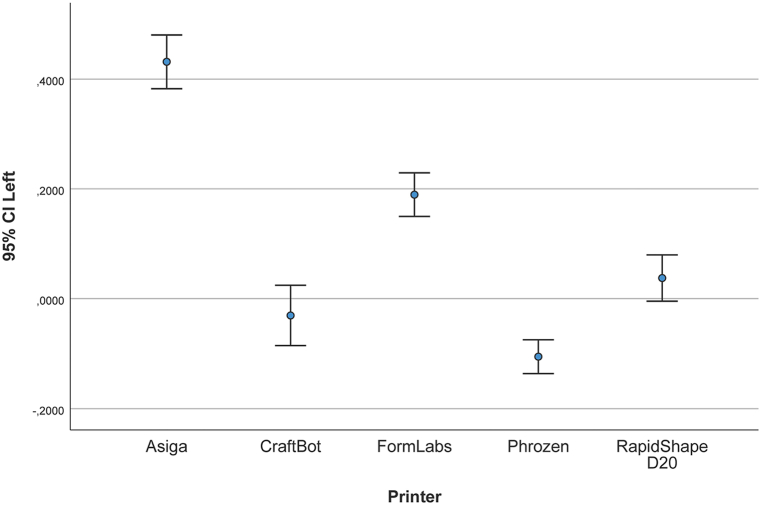
Deviation in distance between right central incisor and left third molar (μm).

Fig. 9 illustrates the mean and SD of the measured distance between the buccal reference points of the upper right central incisor and the third molar. Table 3 shows the linear differences between the test groups and reference data. The mean (SD) was 431.4 (68.2) μm for the AS models, −30.5 (76.7) μm for the CB models, 189.3 (55.5) μm for the FL models, −105.6 (42.9) μm for the PH models, and 37.5 (58.9) μm for the RS models.

**Fig. 10:**
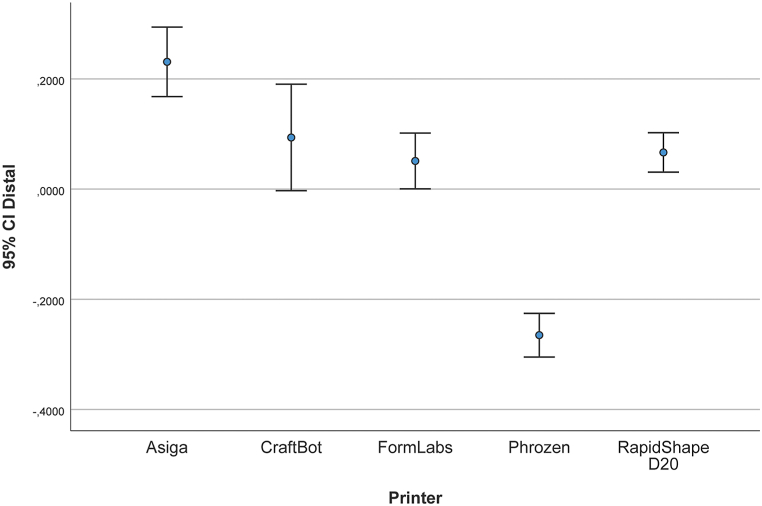
Deviation in distance between upper right third molar and upper left first molar (μm).

Table 3 and Fig. 10 present the data measured between the buccal reference points of the right third and left first molars. The mean (SD) was 231 (88.1) μm for the AS models, 93.6 (135.2) μm for the CB models, 51 (70.9) μm for the FL models, −265.1 (55.4) μm for the PH models, and 66.5 (50.1) μm for the RS models.

The results of the pairwise comparisons of printers determining the significance between the pairs of groups are shown in the Supplementary Material.

## 4 Discussion

The aim of the present study was to investigate the accuracy of five different 3D printing systems, including a comparison of budget- and professional-grade 3D printing systems, according to a standardized production and evaluation protocol. Based on our results, the null hypothesis that there was no significant difference between the models produced by the examined printers was rejected. However, the workflow from a digital file to the final model contains several possibilities for inaccuracies and error, thus limiting the comparability of accuracy outcomes.

Measuring the accuracy of anatomical models is challenging because of their curved shapes, and a suitable reference point cannot always be ensured. Because it was difficult to define clear reference points on the surface of the model for the measurements, 16 half-ball markers were placed on the model for standardization. Using these markers, deficiencies related to the reproducibility of anatomical reference points were resolved [23,37].

Superimposition was used to evaluate the entire deviation of the models. This method is suitable for performing an analysis of the entire surface and assessing dimensional deviations [38]. The mean RMS value for all investigated models was equal to or less than 120 μm with this measurement method. According to a 5-year clinical study, which defined a 120 μm clinically acceptable accuracy limit for fixed restorations [39], these 3D printed models are clinically acceptable. The results showed significant differences from the reference values; however, these were not considered clinically relevant.

Linear measurements with digital calipers can be used efficiently to evaluate the accuracy and reliability of digital dental models [40]. Vertical measurements of the prepared central incisors showed that low-budget printers (CB, PH) can provide the same level of trueness as expensive dental printers. Overall, the trueness values are low, except for the FL model with −153.7 (38.7) μm mean RMS. Several factors can influence the accuracy of printed objects. In particular, the layer height has the greatest effect on the z dimension of the resolution [[41], [42], [43]]. To exclude this element as an influencing factor, all the models were produced with 50 μm layer thickness. This standardization highlights other properties that can cause differences among printers. Unexpectedly, FL showed the worst trueness result. During SLA, the surface of a monomer is scanned using a laser beam [44]. The layer surface is obtained after overlapping photopolymerized strips of a certain width [45]. Based on the overlapping, a more complete polymerization is obtained, and the remaining residual monomer is reduced. This leads to details with precise dimensions and small deformations [45]. Nevertheless, it was unexpected that SLA showed the worst trueness value. This may be due to the fact that the mirror moving the laser beam in the SLA is slow, leading to errors [23].

It was an unexpected result that LCD-based printer PH provided the highest trueness result with −28.2 (23.1) μm measuring the distance between FPD abutments. LCD printing technology has become popular owing to its affordability. This is because the light sources are less expensive than in other printing technologies [46]. LCD panels allow parallel light shining, and lenses or other devices are not required to expand the light. Therefore, the result is not affected by any pixel distortion, leading to high accuracy [47]. Several studies have examined the accuracy of DLP and SLA printers. However, there are few publications on the accuracy of LCD technology. Giudice et al. [48] evaluated the accuracy of dental models produced by entry-level LCD 3D printers compared with SLA-printed models. They concluded that entry-level LCD-based printers are less true and precise than professional-grade SLA printers. Tsolakis et al. [49] compared the accuracy of DLP and LCD printing technologies for dental model printing, and their results suggested that DLP printers are more accurate than LCD printers in the case of dental model printing; however, both printers can accurately be used to print dental models for the fabrication of orthodontic appliances. Another study compared the accuracy of three different printing technologies [50]. They investigated DLP, SLA, and entry-level LCD printers and concluded that the errors of trueness and precision of orthodontic models generated with LCD technology were close to the clinical threshold value (250 μm), suggesting extreme caution with the usage of this type of printer for the production of clear aligners [50]. Compared to the literature results, in our present study measuring FPD abutment deviation, LCD-based PH provided the best accuracy with −28.2 (23.1) μm, SLA-based FL reached 171.7 (33.6) μm, and DLP-based AS and RS showed 165.6 (21.3) μm and 201.9 (41.8) μm mean RMS, respectively.

For linear measurements of the central incisor abutment and abutments for the 4-unit FPD, it was observed that the CB printer shows acceptable mean values (25.9 μm; 111.7 μm) but high standard deviation values (125.3 μm; 82.7 μm). Thus, the 95% confidence interval was wide, indicating a less precise estimate [61]. One disadvantage of filament printers is that the plastic filament material coming out of the circular nozzle tends to shrink, warp, and detach from the platform, causing warp deformation [51]. Moreover, the accuracy of these printers is influenced by its mechanical precision in the x-, y-, and z-axial directions [52]. Because the CB is a mechanical printer, and the head moves in the x- and y-directions to extrude the thermoplastic material, distortion can be observed for long distances [53]. This phenomenon was confirmed by the FPD abutment distance measurement results, in which the standard deviation was high. In this case, the head has to travel between the #14 and #17 abutments, a large distance within a layer, so the shrinkage of the material during the solidification of the two separate material islands can affect the accuracy [53]. This implies that there are differences in accuracy between casts with retained dentition and casts with toothless areas, thus affecting dental use. The maximum distance that could be recorded on the models was between the reference point on the buccal surface of the upper left central incisor and the reference point on the buccal surface of the upper left third molar. In this case, the best trueness was achieved by the CB printer with −30.5 (76.7) μm. This result may be explained by the absence of the need for post-polymerization, as the models had final solidity at the end of the printing process. In contrast, during polymerization-based techniques, the casts must be placed in a cleaning bath and then in a curing unit to achieve the final strength and solidity [20]. During transfer from the printer to the post-processing units, the models can become distorted. This type of deformation is most noticeable when measuring long distances with reduced accuracy. To avoid this phenomenon, a cross-arch support plate on the model can stabilize the shape and reduce shrinkage of the overall volume of the model [54]. This observation is supported by a study in which the accuracy was analyzed in the presence and absence of a cross-arch plate, and the trueness error was found to be significantly larger in the horseshoe-shaped group than in the cross-arch plate group of dental models [31].

Németh et al. [59] concluded in a systematic review and meta-analysis that DLP technology is clinically acceptable for full-arch model production. It was an unexpected result that, in the distance between right central incisor and left third molar and between upper right third molar and upper left first molar, the AS provided high mean differences (231–431 μm). Because DLP printers use a projector instead of a single laser beam to cure the material layer-by-layer, and the process is relatively fast, we expected that errors associated with repeated printing are reduced, and the final result is more accurate compared to other technologies [45]. The reason for these high mean values could be that the range of the measurement dimensions may influence the trueness results [60]. In the accuracy analysis of smaller sections, a section-based alignment method significantly improves the alignment accuracy and decreases the measurement error; therefore, by measuring the deviation in height of the prepared right central incisor and between FPD abutments, we found better trueness results compared to larger distances [60]. Photopolymerization-based technology is much more common in cast fabrication; however, we found that 3D printers using FDM technology can be used for this purpose in terms of trueness [19]. These printers require many more setup parameter settings but at the same time allow for more sensitive fine-tuning, as required by environmental factors and material specifications. The open-source programming of these printer types allows a wider range of development possibilities [53]. It also allows the user's knowledge and practical experience to be better exploited. Unlike resin-based printers, they do not require technician intervention to achieve the final polymerization, which can be advantageous for eliminating the human error factor.

## 5 Limitation

Determining the accuracy of the printing process is challenging because several factors have significant influences. This study aimed to standardize the parameters that can be controlled during production (e.g., layer thickness, model design, and building angle) to properly compare various printers and printed models in terms of accuracy. Despite our defined printing protocol, this study has some limitations. Printing accuracy can also be influenced by the type of material used [62]. The dimensional accuracy is influenced by the composition of the model resin materials and variations in material characteristics, including shrinkage, viscosity, and mechanical properties. These differences can lead to significant discrepancies; therefore, comparability is questionable [63]. Another limitation is that precision was not calculated in this study. However, accuracy can only be fully understood in conjunction with trueness and precision.^36^

## 6 Conclusions

Based on the findings of this in vitro study, the following conclusions were drawn.

1. Budget-level printers provide the same level of accuracy as higher-end 3D printers.
2. FFF printers are acceptable in terms of trueness; however, the dispersion of the results is high.
3. The cost of a printing system is not a reliable indicator of its accuracy.

## Implications for research

Further in vitro and in vivo studies are required to confirm our results, and in vivo studies are required to assess additional 3D printing systems. By increasing the sample size and number of examined 3D printers, reliable guidelines can be established.

A standardized criteria system should be established regarding the parameters and settings of 3D printing systems to reduce confounding factors and enhance the reproducibility and comparability of results. Such a standard criteria system would help manufacturers implement developments tailored specifically for dental use, based on real data.

## Implication for practice

Although accuracy is one of the most important properties of a printing system, there are many other factors to consider. Users should determine the intended use of the 3D printer and choose the appropriate equipment based on their specific needs. Budget-level printers allow users to enter the world of 3D printing without significant investment.

## Ethics approval and consent to participate

Ethical approval was not required for this in vitro study. No patients were involved in the design, conduct, or interpretation of our study.

## Consent for publication

Not applicable.

## Availability of data and material

The authors confirm that the data supporting the findings of this study are available within the article and its Supplementary material. Raw data that support the findings of the study are available from the corresponding author, upon reasonable request.

## Funding

No specific grants were received from funding agencies in the public, commercial, or nonprofit sectors.

## CRediT authorship contribution statement

**Dénes Palaszkó:** Project administration, Investigation, Conceptualization. **Anna Németh:** Writing – original draft, Investigation, Conceptualization. **Gréta Török:** Supervision. **Bálint Vecsei:** Visualization, Supervision, Formal analysis, Data curation, Conceptualization. **Boldizsár Vánkos:** Writing – review & editing, Writing – original draft, Methodology, Investigation. **Elek Dinya:** Validation, Formal analysis, Data curation. **Judit Borbély:** Supervision, Conceptualization. **Gyula Marada:** Conceptualization. **Péter Hermann:** Supervision, Conceptualization. **Barbara Kispélyi:** Validation, Conceptualization.

## Declaration of competing interest

The authors declare that they have no known competing financial interests or personal relationships that could have appeared to influence the work reported in this paper.
