# Supplementary material for: Trueness of five different 3D printing systems including budget- and professional-grade printers: An In vitro study
Source: Heliyon. 2024 Feb 23;10(5):e26874. doi: 10.1016/j.heliyon.2024.e26874 (PMC10925989; doi:10.1016/j.heliyon.2024.e26874)
Supplement: Multimedia component 1 [file mmc1.docx]

**Supplementary materials**

**Detailed description of the printing process**

Each printer was calibrated according to the manufacturers’ instructions (except for the Form 3B, where calibration was not necessary), and the printing parameters (namely: layer height, printing speed, separation pressure limit, material viscosity, material hardness, resolution, slice thickness) were set according to the values coded in the materials’ printing profiles.

The materials were mixed by a mixing machine in group AS; hand-mixed in group PH and group RS, and they required no mixing in group FL, since the Form 3B printer has a built-in material mixer. At each printing site the materials were inspected after mixing, and it was recorded whether air bubbles had formed in them. The ambient temperature at the printing sites was between 19 °C and 22 °C, and the humidity was between 23% and 29%.

**Post-processing of the models**

Models were post-processed according to the manufacturers’ instructions. Group CB (Flow Idex) utilizes no post-processing.

In group AS (Pro4K80) models were washed in an ultrasonic isopropyl immersion cleaning unit - Ultrasonic Cleaner (Soundlin) for six minutes, followed by a water immersion bath - CLD1 (Pro3Dure) for seven minutes. After the washing procedure, the models were air-dried at room temperature with a compressed air duster and were polymerized for 4 minutes at 22 °C using CLD2 (Pro3Dure). The first 40 seconds of the post-curing involved nitrogen insufflation.

In Group FL (Form 3B), models were washed in an isopropyl immersion bath – Form Wash device (Formlabs) for 10 minutes. After the cleaning process, the models were air-dried at room temperature using a compressed air duster. The drying was followed by a 10-minute polymerization at 60 °C using the Form Cure (Formlabs) curing machine.

In group PH (Sonic 4K) models were washed in isopropyl for 10 minutes and polymerized for 8 minutes at 60 °C utilizing a combined washing and curing device – WASH AND CURE 2.0 (Anycubic) (Fig. 1C).

In group RS (D20+) models were washed in an ultrasonic immersion bath – Ultrasonic Cleaner (ASonic) for two minutes, followed by water rinsing. After air-drying with a compressed air duster at room temperature, models were polymerized in PCU LED (Dreve) post-curing device for eight minutes at room temperature.

**Transportation, handling, and storage of the finished printed models**

The 3D-printed models were transported to the laboratory and stored by our workgroup in a hard-shell padded suitcase. This way, they were protected from physical impact and light. Each group had a dedicated storage drawer, where the models were stored spaciously, flat on their base. The ambient temperature of the storage room was between 20 °C and 23 °C and the humidity was between 52% and 60%.
